# Eliciting preferences for continuing medication among adult patients and parents of children with attention‐deficit hyperactivity disorder

**DOI:** 10.1111/hex.13462

**Published:** 2022-03-10

**Authors:** Muhammad Umair Khan, Camila Balbontin, Michiel C. J. Bliemer, Parisa Aslani

**Affiliations:** ^1^ The University of Sydney School of Pharmacy, Faculty of Medicine and Health The University of Sydney Sydney New South Wales Australia; ^2^ Aston Pharmacy School, College of Health and Life Sciences Aston University Birmingham United Kingdom; ^3^ Institute of Transport and Logistics Studies, The University of Sydney Business School The University of Sydney Sydney New South Wales Australia

**Keywords:** ADHD, adults, discontinuation, implementation, medication, parents, preferences

## Abstract

**Background:**

Adherence to medication for attention‐deficit hyperactivity disorder (ADHD) is less than optimal. Previous studies have primarily focused on qualitative assessment of factors that influence medication adherence.

**Objective:**

This study aimed to quantify the factors that influence patient and parent preferences for continuing ADHD medication.

**Method:**

A discrete‐choice experiment was conducted to investigate preferences. Adults, and parents of children, with ADHD were presented with eight hypothetical choice tasks of three options (Medication A, Medication B, No Medication) described by six attributes related to medication outcomes. Preferences were estimated using a mixed multinomial logit model.

**Results:**

Overall, respondents' preferences (*n* = 216) for continuing medication were negative (mean [*β*] = −1.426, *p* < .001); however, a significant heterogeneity in preferences was observed amongst respondents (standard deviation = 0.805, *p* < .001). Improvements in education, aggressive behaviour, social behaviour and family functioning, and side effects and stigma, influenced respondents' decision to continue taking medication. The respondents were willing to continue medication if they experienced positive effects, but side effects (even moderate) were the strongest concern for not continuing medication. While side effects were the most important factor for both adult patients and parents of children with ADHD, improvement in education was relatively more important for adults and improvement in aggressive behaviour, social behaviour and family functioning was relatively more important for parents of children with ADHD. Parents were more likely to not continue a medication with severe side effects even at the highest level of improvement in education.

**Conclusions:**

Side effects are the most important factor that influenced preferences for continuing medication for both adults with ADHD, as well as parents of children with ADHD. While overall the respondents preferred not to take/give medication, discrete‐choice experiment showed that the relative importance of factors that influenced continuation of medications was different for the two groups.

**Patient and Public Involvement:**

Adults, and parents of children, with ADHD participated in this study by completing the online questionnaire. The questionnaire was based on findings of research in the literature, as well as earlier focus groups conducted with adults, and parents of children, with ADHD. The face validity of the questionnaire was determined by asking parents of children, and adults, with ADHD (*n* = 3) to complete the survey and participate in a short discussion on their understanding of the questions and their recommendations on improving the clarity of the survey.

## 1 BACKGROUND

Attention‐deficit hyperactivity disorder (ADHD) is defined as a persistent pattern of inattention, hyperactivity and/or impulsivity that interferes with an affected person's daily functioning.^1^ ADHD is a common neurodevelopmental disorder among children, with a worldwide prevalence of 7.2%.^2^ The prevalence of ADHD in adults is between 1.2% and 7.3%^3^; however, it has been suggested that these estimates are not accurate as only one‐quarter of adults living with ADHD are diagnosed.^4^, ^5^, ^6^ ADHD can have a significant impact on the academic, occupational and social life as well as family relationships of those affected.^7^ ADHD also has a significant economic impact and is associated with higher healthcare costs. The annual cost of raising children and adolescents with ADHD in the United States (US) has been estimated as US$124.5 billion.^8^ Similarly, the estimated healthcare and productivity cost associated with adults with ADHD ranged between US$87 billion and US$138 billion in the US in 2010.^9^

Given the breadth of impact, management of ADHD is important in achieving better health and social outcomes for those affected. Pharmacotherapy is an important component of the overall management and is recommended by various international guidelines.^10^, ^11^, ^12^ These guidelines suggest that long‐term use of medication is important to achieve the desired medication outcomes; however, evidence shows that patients do not adhere well to medication^13^ and often discontinue medication within the first few months of therapy.^14^ Adherence to medication is defined as the process by which patients take their prescribed medication and is composed of initiation, implementation and discontinuation as well as persistence.^15^

Numerous factors can influence medication adherence in people with ADHD.^14^, ^15^, ^16^, ^17^, ^18^, ^19^ Lack of medication effectiveness, side effects, stigma and high costs have primarily contributed to the higher rates of nonadherence.^13^, ^20^, ^21^ While there is some information about how these factors impact adherence,^22^ there are very limited quantitative data on the relative importance (RI) of these factors. Initiation is defined as the consumption of the first dose of a prescribed medication. Implementation is defined as the continuation of medication as prescribed. Discontinuation is defined as the cessation of a prescribed medication.^15^ Once initiated, people with ADHD can struggle with their decision‐making and often stop and restart medication multiple times through their medication‐taking journey.^23^, ^24^ Identifying adherence phase‐specific factors and their relative importance is central to designing targeted interventions, as factors influencing adherence vary in their impact on different phases of medication‐taking.^22^, ^25^, ^26^

Adherence research in ADHD has used sociobehavioural models to explain the complex phenomenon of adherence.^27^, ^28^, ^29^, ^30^, ^31^ However, there is no clear evidence that these models can help in designing interventions to promote adherence that are effective and sustainable.^7^ Furthermore, there is limited research investigating the choice that people make between various medication attributes, such as benefits, side effects and cost, to decide on medication adherence. Patients continuously make a conscious decision to adhere/not adhere to medication by valuing the medication and its outcomes.^32^ The valuation or utility can be estimated through a discrete‐choice experiment (DCE). DCEs have been widely used for eliciting preferences in health research.^33^ DCEs can assist in understanding patients' trade‐off between benefits and concerns about medication, which in turn influence their decision to adhere/not adhere to medication.^34^ Furthermore, DCEs can investigate attributes that may influence participants' decision to continue medication in addition to their willingness to continue or discontinue medication. DCEs often involve stated choice data where individuals are presented with hypothetical choice tasks and asked to express a preference based on their experiences.

In the current literature, decision‐making about ADHD medication has been primarily explored through qualitative research suggesting a number of factors that impact preferences for medication.^21^, ^22^, ^23^, ^24^, ^25^, ^26^ However, little effort has been made to quantify these factors and investigate which factors are more important than others. Understanding the relative importance of the factors is important in prioritizing interventions to improve medication adherence and its outcomes. In particular, as noted above, it is important to assess the importance of factors at each of the three phases of medication‐taking to develop tailored interventions.^25^ A few studies have used DCEs to assess preference for medication in people with ADHD.^35^, ^36^, ^37^, ^38^ These studies have shown that the benefits of medication, particularly improvement in behaviour and social situations, were the most preferred attributes for patients and their carers. However, none of the previous studies examined medication attributes in the context of the three phases of medication adherence. Moreover, previous studies were either limited to a class of medication, such as stimulants,^38^ or a population, such as parents of children,^37^ children^36^ or adults with ADHD.^35^ Therefore, the objective of this study was to investigate preferences for continuing ADHD medication based on key factors shown to influence adherence to ADHD medication, and determine the relative importance of those factors, in parents of children and adults with ADHD.

## 2 METHODS

### 2.1 Study participants and recruitment

Participants were recruited by advertising the research on online platforms such as Facebook. The invitation was advertised on various Australian‐based ADHD support groups on Facebook and also on their websites. By using the ‘Boost’ option (available on Facebook), the invitation was customized to the target audience by predefining their location (Australia), age (18–65 years) and interest (ADHD, managing a child with ADHD, ADHD awareness). Potential participants were provided with an online link to access the anonymous survey. However, eligibility screening was performed first, which ensured that only eligible participants could access the survey.

### 2.2 Study instrument

A web‐based survey was conducted with parents of children, and adults, with ADHD in Australia. The survey was divided into three parts. The first part screened the respondents for their eligibility to participate. The second part involved choice‐based questions (DCE) in which eligible respondents were asked to choose their preferred hypothetical medication (or no medication) from a set of alternatives based on given attributes and attribute levels. The third part asked questions about participants' characteristics such as, age, gender and income.

#### 2.2.1 Part 1: Eligibility criteria

The inclusion criteria were (1) parent of a child with ADHD aged up to 17 years, or an adult (18–65 years) diagnosed with ADHD, irrespective of when the diagnosis was made (as a child or as an adult), and (2) taking prescribed medication or giving it to their child for ADHD. Participants were only recruited if they were based in Australia. The survey was conducted in English.

#### 2.2.2 Part 2: Choice‐based survey

The choice‐based survey was conducted using a DCE.^36^ DCEs provide a systematic method of quantifying trade‐offs and the importance that a person assigns to a set of treatment attributes to decide whether they prefer a particular treatment, such as, medication. Each attribute (e.g., medication side effects) has various attribute levels (e.g., mild, moderate, severe) that describe the dimensions of that attribute. Each participant was provided with eight hypothetical choice tasks. In each choice task, participants were asked to choose their preferred medication (Medication A or Medication B) or a ‘no medication’ option (I will not continue either Medication A or Medication B). Medications A and B differed in terms of attribute levels. Those who chose the ‘no medication’ option were provided with a supplementary question which asked that if they had to choose between Medication A or B, which one would they prefer to continue. The supplementary question was asked to encourage people to make trade‐offs between different treatment attributes. The underlying theory of DCEs, the random utility theory, assumes that individuals are rational in making their decision and choose the alternative that maximizes their utility function. The utility function of each alternative is defined by its attribute levels and individuals' valuation of each parameter estimate (*β*). Therefore, the first step in designing the survey was to select a set of attributes and attribute levels to describe each alternative that was relevant to the study population.

A review of the literature was conducted to identify the factors that influence adherence to ADHD medication at the implementation and discontinuation phases of adherence.^39^ This was followed by focus group discussions (FGs) with people with ADHD and parents of children with ADHD to further identify factors influencing adherence.^21^, ^40^ Identified factors were discussed amongst authors for their potential inclusion in the DCE. Six attributes that were consistently identified as important in the literature and the FGs were selected: (1) improvement in education and learning; (2) improvement in aggressive behaviour; (3) improvement in social behaviour; (4) improvement in family functioning; (5) severity of side effects; and (6) presence of social stigma. The first four attributes had three levels (no improvement, somewhat improved, considerably improved), the fifth attribute had four levels (none, mild, moderate, severe) and the last attribute had two levels (present or not present) (Table 1). The attribute levels were chosen based on the literature^39^ and the FGs^21^, ^40^ to maximize trade‐off and improve the reliability of parameter estimates.

**Table 1:** Factors influencing continuation of medication—attributes and levels

Attributes	Levels	Description^a^	Expected sign
Improvement in education and learning	No improvement	Education and learning have not improved at all. Rating = 0/10	0 Dummy variable base
	Somewhat improved	Education and learning have improved to some extent, but ADHD still affects your/your child's learning. Rating = 4/10	+
	Considerably improved	Education and learning have improved considerably, and ADHD does not affect your/your child's learning. Rating = 8/10	++
Improvement in aggressive behaviour	No improvement	Aggressive behaviour has not improved at all. Rating = 0/10	0 Dummy variable base
	Somewhat improved	Aggressive behaviour has improved to some extent with medication, but ADHD still affects your/your child's behaviour. Rating = 4/10	+
	Considerably improved	Aggressive behaviour has improved with medication and ADHD does not affect your/your child's behaviour. Rating 8/10	++
Improvement in social behaviour	No improvement	Social behaviour has not improved at all. Rating = 0/10	0 Dummy variable base
	Somewhat improved	Social behaviour has improved to some extent with medication, but ADHD still affects your/your child's social interactions. Rating = 4/10	+
	Considerably improved	Social behaviour has improved with medication and ADHD does not affect your/your child's social interactions. Rating = 8/10	++
Improvement in family functioning	No improvement	Family functioning has not improved at all. Rating = 0/10	0 Dummy variable base
	Somewhat improved	Family functioning has improved to some extent with medication, but ADHD still affects your/your family functioning. Rating = 4/10	+
	Considerably improved	Family functioning has improved with medication, and ADHD does not affect your/your family functioning. Rating = 8/10	++
Medication's side effects	None	There are no side effects	0 Dummy variable base
	Mild	Side effects do not interfere with your/your child's daily routine (such as exercise, work, education, family/leisure)	–
	Moderate	Side effects interfere with your/your child's daily routine (such as, exercise, work, education, family/leisure), but you/your child can still perform routine activities	–
	Severe	Side effects interfere with your child's daily routine (such as, exercise, work, education, family/leisure) and you/your child is not able to perform routine activities	–
Social stigma with medication	Present	People treat you/your child differently when your child takes medication	–
	Not present	People do not treat you/your child differently when your child takes medication	0 Dummy variable base

Abbreviation: ADHD, attention‐deficit hyperactivity disorder.

^a^ Rating scale: 0 indicates no improvement and 10 indicates exceptional improvement.

**Figure 1:**
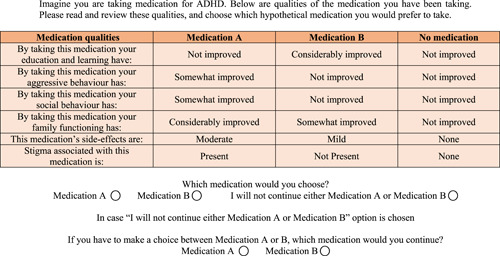
Example choice task. ADHD, attention‐deficit hyperactivity disorder

The next step was to create an experimental design using Ngene software. An unlabelled experiment was designed as the objective of this study was not to assess preferences for specific medications used in ADHD, but to assess the factors that influence a decision of whether or not to continue with the medication. Given the large number of possible choice sets (*n* = 419,904), a full‐factorial design was not feasible. Therefore, a fractional‐factorial design was used, and a subset of 24 possible choice tasks was selected by generating an attribute level‐balanced D‐efficient design (Appendix S1). Efficient designs have the potential to draw a subset that can yield more information, produce smaller standard errors and increase the reliability of the parameter estimates.^41^ An important consideration in using efficient designs is the use of prior parameter estimates. However, it is also suggested that if the priors are not correct or close to actual behaviours, the efficient design can easily become inefficient.^42^ Given that no priors were available from the literature, we set very small prior values, almost equal to zero, with the expected signs as predicted in Table 1. Further, we ensured that no dominant alternatives were present in our survey.^43^ Eight choice tasks, consistent with previous health research,^44^, ^45^ were deemed appropriate for each participant, which means that we used three blocks for our design. An example of a choice task is illustrated in Figure 1. All participants considered each individual level of the six attributes across the choice sets at least once. Before administration, the survey was pretested for face and content validity. Content validity was first determined by asking experts (researchers, subject experts) to review the survey and provide suggestions on improving the content of the survey in line with the study objectives. Face validity was determined by randomly selecting parents of children, and adults, with ADHD (*n* = 3) and asking them to complete the survey and participate in a short discussion on their understanding of the questions and their recommendations on improving the clarity of the survey. Minor modifications were made to the survey, leading to improved clarity. In the final survey, participants were provided detailed information about the choice survey and its objectives, as well as a detailed description of attributes and levels. Furthermore, a practice choice task was also given to the participants before the actual choice tasks (Figure 1). Since priors were not known, it was not possible to estimate the required sample size. Rose and Bliemer^42^ suggest that the most practical approach in such a situation is to maximize the efforts to increase sample size within the available time and budget.

#### 2.2.3 Part 3: Participants' characteristics

The last section of the survey asked demographic questions such as age, gender, education, employment and other questions related to participants' past and current ADHD treatment.

### 2.3 Data analysis

Data were analysed using NLOGIT version 6.0 to estimate a mixed multinomial model (MMNL).^46^ McFadden's pseudo *R* ^2^ was used to evaluate the model's goodness of fit. The MMNL was chosen because of its potential to account for the heterogeneity in preferences among respondents by considering the panel nature of the data (multiple observations by a single respondent), which was essential to assess the complexities of adherence. All attributes and sociodemographic variables were dummy‐coded, except for age, which was retained as a continuous variable. The final model was selected based on the Akaike information criterion (AIC), which is a widely used criterion to decide which variables to include and exclude. AIC is defined as 2*k−2* ln(*L*), where *k* is the number of parameters in the model, *n* is the number of data points and *L* is the final log‐likelihood function. We inserted all attributes in the choice tasks as main effects into the model and added only (combinations of) covariates and interaction effects if they lower the AIC. Parameter estimates (including the intercept) with a significant standard deviation were retained as normally distributed random parameters, while the remaining parameters were considered fixed. Halton draws (*n* = 500) were used to estimate the final model (Appendix S2). The parameter estimates (mean = *β*) indicated the importance of attributes with higher estimates representing higher utility for an attribute.

The model also quantified the relative importance (RI) of each attribute. The RI of attributes is useful to describe how much difference each attribute could make in the overall utility of an alternative. The RI of attribute *k* was obtained by computing its impact on utility, expressed as the range *X*_k_ between the highest part‐worth and lowest part‐worth utility, and then dividing by *Σ*_k_ *X*_k._ The RI of an attribute was calculated by dividing the utility range of that attribute by the total utility.^47^ The utility range of an attribute was obtained by subtracting the lowest part‐worth utility of that attribute from its highest part‐worth utility, whereas the total utility was obtained by adding the utility range across the attributes. RI was presented as percentages. Subgroup analysis was conducted, and the RI was calculated for both parents of children, and adults, with ADHD. Both adult and parent samples were combined in a single model as well as their interaction with other attributes. Estimation of a joint model using the pooled sample where interaction effects are included between the attributes and the group dummy variable is a common practice in choice analysis. This approach not only allows preferences to be different across the two groups, but we can also easily test whether preferences are statistically different across the two groups by assessing the statistical significance of the interaction coefficients. The demographic information of the respondents was included in the model as independent variables, and as interactions with the attributes. The coefficients obtained from the interactions (between group and medication attributes) were used to calculate the RI of attributes between the sub‐groups.

Simulation analysis or elasticity calculations were also conducted to investigate the change in the probability of continuing medication with the change in the levels of the attributes (Appendix S3). We examined different levels of side effects (mild, moderate, severe) against different levels of education (somewhat improved, considerably improved) and determined the change in the probability of continuing medication if one level changes to another. For example, if the side effects are mild, what would be the probability of continuing medication if the education level changes from no improvement to somewhat improvement. All levels of side effects were simulated for somewhat improvement and considerable improvement in education and learning. The basic multinomial model was first estimated, and then the simulation commands were specified in the model (Appendix S3), which yielded the probabilities of continuing medication for the given scenarios.

### 2.4 Ethics approval

This study was approved by the University of Sydney's Human Research Ethics Committee (project no. 2019/429). A participant information sheet was provided to the respondents before the survey that included detailed information about the research and its objectives. Participation in this study was voluntary, and the submission of the survey was taken as participants' consent to participate.

## 3 RESULTS

### 3.1 Participants' characteristics

Two hundred and sixteen respondents completed the survey. One hundred and twenty‐eight respondents were adults with ADHD (59.3%) and eighty‐eight respondents were parents of children with ADHD (40.7%). The characteristics of the parents and adults are presented in Table 2.

**Table 2:** Participants' characteristics (*n* = 216)

Variable	Parents^a^ (*n* = 88)	Adults (*n* = 128)	Total sample (*n* = 216)
	Mean (SD)	Mean (SD)	Mean (SD)
Age (years)	40.9 (9.2)	33.6 (12.3)	36.6 (11.7)
Age of respondents' child with ADHD (years)	10.2 (4.7)		
	Number (%)	Number (%)	Number (%)
Gender			
Male	9 (10.2)	40 (31.3)	49 (22.7)
Female	79 (89.8)	83 (64.8)	162 (75)
Missing	0	5 (3.9)	5 (2.3)
Annual income (AUD)			
<52,000	26 (29.5)	35 (27.3)	61 (28.2)
52,000–129,000	30 (34.1)	53 (41.4)	83 (38.4)
>130,000	32 (36.4)	25 (19.5)	57 (26.4)
Missing data	0	15 (11.8)	15 (7)
Education			
School, year 10	5 (5.7)	3 (2.3)	8 (3.7)
School, year 11–12	5 (5.7)	18 (14.1)	23 (10.6)
Diploma	16 (18.1)	38 (29.7)	54 (25.0)
Bachelor's degree and higher	62 (70.5)	68 (53.1)	130 (60.2)
Missing data	0	1 (0.7)	1 (0.4)
Marital status			
Single	10 (11.3)	55 (42.9)	65 (30.1)
De facto	8 (9.1)	26 (20.3)	34 (15.7)
Married	58 (65.9)	30 (23.4)	88 (40.7)
Divorced	12 (13.6)	15 (11.8)	27 (12.5)
Missing data	0	2 (1.6)	2 (0.9)
Employment			
Employed	68 (77.3)	90 (70.3)	158 (73.1)
Not employed	20 (22.7)	34 (26.6)	54 (25.0)
Missing data	0	4 (3.1)	4 (1.9)
Health‐related employment			
Yes	30 (34.1)	36 (28.1)	66 (30.6)
No	58 (65.9)	90 (70.3)	148 (68.5)
Missing data	0	2 (1.6)	2 (0.9)
Current medication for ADHD			
Methylphenidate (short‐acting)	20 (22.7)	27 (21.1)	47 (21.8)
Methylphenidate (long‐acting)	21 (23.9)	3 (2.3)	24 (11.1)
Methylphenidate (extended‐release)	29 (33)	13 (10.2)	42 (19.4)
Atomoxetine	9(10.22)	10 (7.81)	19 (8.8)
Guanfacine	17 (19.3)	2 (1.6)	1 (0.5)
Lisdexamphetamine	17 (19.3)	21 (16.4)	38 (17.6)
Dexamphetamine	6 (6.8)	48 (37.5)	54 (25)
Clonidine	5 (5.7)	0	5 (2.3)
Past medication for ADHD			
Yes	76 (86.4)	96 (75)	172 (79.6)
No	12 (13.6)	31 (24.2)	43 (19.9)
Missing data	0	1 (0.8)	1 (0.5)
Current nonpharmacological therapy for ADHD			
Yes	43 (48.9)	58 (45.3)	101 (46.8)
No	45 (51.1)	70 (54.7)	115 (53.2)
Type of nonpharmacological therapy for ADHD^b^			
Behavioural modification	13 (14.8)	17 (13.3)	30 (13.9)
Cognitive behavioural therapy	23 (26.1)	39 (30.5)	62 (28.7)
Anger management	6 (6.8)	3 (2.3)	9 (4.2)
Social therapy	14 (15.9)	5 (3.9)	19 (8.8)
Family therapy	10 (11.4)	7 (5.5)	17 (7.9)
Speech therapy	12 (13.6)	18 (14.1)	30 (13.9)
Occupational therapy	4 (4.5)	1 (0.8)	5 (2.3)
Past nonpharmacological therapy for ADHD			
Yes	58 (65.9)	77 (60.2)	135 (62.5)
No	30 (34.1)	51 (39.8)	81 (37.5)
Self‐control			
High	42 (47.7)	74 (57.8)	116 (53.7)
Low	44 (50)	51 (39.8)	95 (44)
Missing data	2 (2.2)	3 (2.3)	5 (2.3)
Concomitant conditions			
Yes	62 (70.5)	102 (79.7)	164 (75.9)
No	26 (29.5)	26 (20.3)	52 (24.1)
Concomitant conditions^b^			
Autism spectrum disorder	31 (35.2)	17 (13.3)	48 (22.2)
Learning disabilities	17 (19.3)	16 (12.5)	33 (15.3)
Oppositional defiance disorder	16 (18.2)	4 (3.1)	20 (9.3)
Anxiety	39 (44.3)	84 (65.6)	123 (56.9)
Depression	6 (6.8)	61 (47.7)	67 (31.0)

Abbreviation: ADHD, attention‐deficit hyperactivity disorder.

^a^ Data on current medication, past medication, current nonpharmacological therapy, past nonpharmacological therapy, self‐control and concomitant conditions are for children reported by their parents.

^b^ More than one option was chosen by respondents.

### 3.2 DCE results

#### 3.2.1 The mixed multinomial logit model

Overall, respondents' preferences to continue taking medication were negative (mean = *β* = −1.426, *p* < .001). Significant heterogeneity was noted in respondents' (adults and parents) preferences for continuing medication. All attributes, improvements in education, aggressive behaviour, social behaviour and family functioning, and side effects and stigma, significantly influenced respondents' decision to continue taking medication. As expected, improvement in education, aggressive behaviour, social behaviour and family functioning significantly increased utility or preference for medication, while side effects and stigma had a negative influence on the preference for medication. It was noticed that the preference for continuing medication increased with the increase in the level of improvement of education, aggressive behaviour, social behaviour and family functioning. For example, the preference for continuing medication was higher with considerable improvement in education (*β* = 3.627, *p* < .001) compared to somewhat improvement in education (*β* = 2.359, *p* < .001). In contrast, and as expected, the preference for medication decreased with the increase in the severity of side effects and the presence of stigma. For example, the preference for medication was lower with severe side effects (*β* = −5.015, *p* < .001) compared to moderate (*β* = −.891, *p* < .001) and mild (*β* = −.358, *p* < .05) side effects. The parameter estimates are presented in Table 3.

**Table 3:** Mixed multinomial logit model output

Attributes	Coefficient	Confidence interval	Standard error
Medication (constant)^a^—mean	−1.426***	−0.992 to −1.859	0.221
Medication (constant)^a^—SD	0.805***	0.546 to 1.061	0.132
Scaling parameter	−0.648***	−0.521 to −0.774	0.064
Education			
No improvement	Base		
Somewhat improvement	2.359***	2.05 to 2.666	0.157
Considerable improvement^a^—mean	3.627***	3.254 to 3.999	0.190
Considerable improvement^a^—SD	0.608***	0.363 to 0.853	0.125
Aggressive behaviour			
No improvement	Base		
Somewhat improvement	0.686***	0.340to1.031	0.208
Considerable improvement^a^—mean	1.036***	0.673 to 1.398	0.185
Considerable improvement^a^—SD	0.338*	−0.032 to 0.708	0.189
Social behaviour			
No improvement	Base		
Somewhat improvement	1.356***	1.056 to 1.655	0.153
Considerable improvement^a^—mean	1.548***	1.275 to 1.820	0.139
Considerable improvement^a^—SD	0.235	−0.141 to 0.611	0.192
Family functioning			
No improvement	Base		
Somewhat improvement	0.585***	0.281to 0.888	0.155
Considerable improvement^a^—mean	1.124***	0.816 to1.431	0.157
Considerable improvement^a^—SD	0.275	−0.066 to 0.616	0.174
Side effects			
No side effects	Base		
Mild	‐0.358**	−0.695 to 0.020	0.172
Moderate^a^—mean	−0.891***	−1.24 to 0.534	0.182
Moderate^a^—standard deviation	1.035***	0.693 to 1.376	0.174
Severe^a^—mean	−5.015***	−5.785 to −4.244	0.393
Severe^a^—standard deviation	3.255***	2.512 to 3.997	0.379
Stigma			
Not present	Base		
Present	−0.253**	−0.468 to −0.037	0.110
Other variables (interaction effects)			
Group × education − somewhat improvement	−0.887***	−1.335 to −0.438	0.229
Group × education − considerably improvement	−0.947***	−1.372 to −0.521	0.217
Group × behaviour^b^ − somewhat improvement	0.668***	0.181 to 1.1540	0.248
Group × behaviour^b^ − considerably improvement	0.667***	0.177 to 1.157	0.250
Group × side effects − severe	−1.766***	−2.808 to −0.723	0.532

*Note*: Coefficients in bold number fonts were found to be significant after applying the Bonferroni correction at a significance level of 5%.

Log‐likelihood function = −1180.140.

Pseudo *R* ^2^ = 1‐(−1180.140/−2192.830) = 0.462.

Inf.Cr.AIC (Akaike information criterion) = 2412.3.

Group: 1 = parents; 0 = adults. Total number of observations = 1996.

***, **, *Significance at 1%, 5%, 10% level, respectively.

^a^ Random parameters.

^b^ Behaviour = aggressive behaviour.

It was also observed that the decision to continue medication varied between parents and adults with respect to various medication attributes. For example, adults were more likely to continue medication if there was an improvement in the level of education (somewhat improvement: *β* = 2.359; considerable improvement: *β* = 3.627) compared to parents (somewhat improvement: *β* = 1.472; considerable improvement: *β* = 2.68). However, improvement in aggressive behaviour was less important to adults (somewhat improvement *β* = .686; considerable improvement *β* = 1.036), that is, they were less likely to continue medication if there was an improvement in aggressive behaviour than parents (somewhat improvement: *β* = 1.354; considerable improvement: *β* = 1.703, *p* < .001). While no significant difference was observed between parents' and adults' preferences in case of mild and moderate side effects, parents were less likely to continue giving medication to their child compared to adults in the event of severe side effects (parents *β* = −6.781, adults *β* = −5.015).

### 3.3 Relative importance of attributes

**Figure 2:**
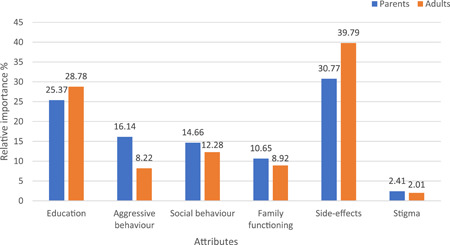
Relative importance of attributes between parents and adults

Overall, both parents and adults considered side effects as the most important attribute in their decision‐making about the continuation of medication. The RI of side effects for parents and adults was 30.77% and 39.79%, respectively. The next important factor was improvement in education (RI = 25.37% for parents; 28.78% for adults), while stigma was the least important factor in both groups (RI = 2.41% for parents and 2.01% for adults). Improvement in social behaviour (RI = 14.66% vs. 12.28%), aggressive behaviour (RI = 16.14% vs. 8.22%) and family functioning (10.65% vs. 8.92%) was relatively more important to parents than adults with ADHD (Figure 2).

### 3.4 Simulated probabilities

**Figure 3:**
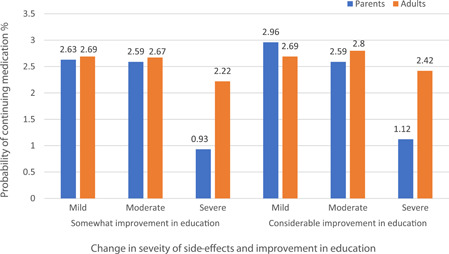
Change in the probability of continuing medication with change in the severity of side effects and improvement in education

The simulation findings showed that both parents and adults were more likely to continue taking medication if they were presented with mild levels of side effects compared to moderate and severe levels. At any given level of side effect (mild, moderate or severe), the probability of continuing medication was higher when there was considerable improvement in education compared to somewhat improvement in education. The probability of continuing medication was similar between parents and adults when the side effects were mild and moderate; however, a notable difference in preference was observed between the two groups when side effects were severe. Parents were less likely to continue giving medication to their child compared to adults when either the improvement in education was somewhat improved (probability of continuing medication 0.93% for parents vs. 2.22% for adults) or considerably improved (1.12% for parents vs. 2.42% for adults; Figure 3).

## 4 DISCUSSION

The findings of this study showed negative preferences for continuing medication in parents of children and adults with ADHD. That is, they preferred not to continue with the medication. However, significant heterogeneity existed in their preferences as shown by the results of the mixed logit model, which revealed a statistically significant standard deviation. The decision to continue medication was influenced by several factors, and the impact of those factors was relatively different for different people. The current literature on decision‐making about ADHD medications has primarily focused on qualitative research, which suggests that decision‐making is influenced by myriad of factors (such as side effects and improvement in symptoms)^16^, ^17^, ^18^, ^19^, ^20^, ^21^; however, this study quantified the preferences and adds to the literature that side effects are the most influential factor that impacts decision‐making in both adults and parents of children with ADHD. This might be the reason why the overall preferences for continuing medication were negative in this study. Furthermore, this study adds to the literature by demonstrating that improvements in aggressive behaviour, social behaviour and family functioning were more important to parents of children with ADHD compared to adults with ADHD. Another novel finding of this study was that preference for both parents and adults to continue medication was similar, with mild and moderate levels of side effects for any level of improvement in education; however, parents were more likely to not continue medication with severe side effects even at the highest level of improvement in education.

Our findings demonstrated that people with ADHD considered several factors in making decisions about medication. These factors are predominantly related to medication outcomes, positive (improvement in symptoms) or negative (side effects). Preference for medication increased with the positive outcomes and decreased with the negative outcomes. However, decision‐making about ADHD medication is not straightforward, but rather, a balancing act between positive and negative outcomes that people perform throughout the process of medication‐taking. This study confirms previous reports that various factors influence decision‐making about ADHD medication,^7^, ^21^, ^40^ and further identifies that different factors have different impacts on decision‐making and that the impacts also change between different groups. These findings may be better understood through the lens of the Necessity‐Concerns Framework (NCF). According to the NCF, adherence to medication depends on necessity beliefs and concerns about medication.^48^ If the necessity beliefs are higher than concerns, a patient is more likely to take medication. The beliefs and concerns are highly heterogeneous, that is, every individual may have his/her own beliefs and concerns about medication. For example, previous studies have shown when people (with the same medical condition) are prescribed the same medication, they differ in their perceptions of personal need for it.^49^ Some people may consider medication a necessity, while others may not. This perception is further influenced by concerns such as side effects. For example, a patient who is experiencing side effects may have a negative perception of medication and therefore may not take medication despite the physician's recommendation. Against this background, the current study findings suggest that necessity beliefs (improvement in education, aggressive behaviour, social behaviour, family functioning) positively impact the need for medication, and concerns (side effects, stigma) have a negative impact, and the ultimate decision to continue the medication rests on the balance between necessity and concerns.

Previous studies have reported that perceived medication effectiveness and side effects are the main two factors that contribute to the decision‐making about ADHD medication.^50^, ^51^ The present study extends this study by demonstrating that side effects are relatively more important than improvement in education or behaviour and have a higher impact on the necessity–concerns balance, and hence, increase the likelihood of medication discontinuation. This study further adds that people are more willing to continue medication with mild and moderate side effects, but less for severe side effects to gain improvements in education and behaviour. Thus, people make a conscious decision to initiate medication considering that medication maximizes their utility^22^, ^40^; however, soon after initiation, they start to experience, and balance, the positive (effectiveness) and negative (side effects, stigma) effects of medication. The balanced point, however, varies across the population. For example, one person may continue to take medication despite having mild side effects because of the benefits of medication, while another person may find the same side effects (with similar severity) hard to tolerate despite experiencing the benefits of medication. Furthermore, some people may be willing to accept side effects for improvement in education, while others may consider social factors more important than education, and hence make their decision accordingly. These arguments are also supported by previous research showing significant heterogeneity in the preferences of the people with ADHD and parents of children with ADHD.^52^

Another important contribution of this study is that it highlights the difference in the impact of factors, particularly aggressive behaviour and social behaviour, on decision‐making between parents and adults. Our findings suggest that improvement in social behaviour and aggressive behaviour is more important to parents compared to adults with ADHD. We offer several possible explanations for the difference in the impact of factors. First, parents are generally more concerned about the aggressive behaviour of their child whose inability to recognize risk can put his/her and others' lives in danger.^22^ Parents are also concerned that their child may hurt themselves or others unintentionally or may get expelled from school due to aggressive behaviour. These concerns may lead to psychological distress and lower sense of parenting competence, hence why they consider improvement in aggressive behaviour important. Furthermore, adults may be better at controlling aggressiveness compared to children.^53^ Second, parents may be more concerned about the future of their children in terms of how they will cope with the wider social challenges of life such as educational, occupational, marital and interpersonal communication issues. Parents may believe that over time, these challenges may lead to negative social outcomes and social rejection of people with ADHD; hence, improvements in social behaviour may become more important to parents. Third, ADHD in adults, compared to children, is more likely to be associated with false beliefs, negative attitudes and lack of knowledge/understanding of ADHD among the public and health professionals, which might lead to self‐ as well as public stigmatization of an individual with ADHD.^54^ These explanations, therefore, support the notion that the motivation to continue medication could be different for parents and adults.

Attributes were selected through a rigorous three‐step process that included a comprehensive review of the literature,^39^ FGs^22^, ^40^ and discussion among authors to finalize a set of attributes to be included in the study. The pre‐testing of the survey with both parents and adults confirmed the relevance of the selected attributes in the medication‐taking decision‐making. The rigorous process for selecting the attributes is a major strength of this study as it makes the findings more applicable to the clinical and research settings, particularly for the identification of patients' and parents' preferences for medication and the development of interventions to improve medication adherence.

### 4.1 Limitations

The findings of this study should be interpreted within the context of some limitations. One of the main challenges in our research was in the recruitment of the right respondents to answer our survey: adults and parents. Therefore, one of the limitations of this study is our sample size. This was overcome by pooling both data sets and using interactions to identify differences in each group. However, the complexity of our model in terms of interactions and random parameters was limited by the sample size to ensure that the model was correctly estimated. However, the statistical significance of several attributes in the model suggests that lack of power was not a concern. Given the online nature of data collection, it was not possible to determine the response rate. Second, the part worth estimates and the RI were calculated for the parents and the adult groups with relatively different sample sizes (adults = 128; parents = 88), which may have an impact on parameter estimates. Furthermore, any changes to the number of attributes or their parameter estimates might change the RI of the attributes for the two groups. Third, six attributes were chosen for the DCE. While the attributes were chosen and defined based on the literature review and FGs, consistent with previous suggestions, there is a possibility that those attributes may not fit well to all people with ADHD and their carers, which may limit the generalizability of the findings. However, choice experiments have to find the right balance between the number of attributes, their descriptions and burden on respondents, particularly in people with ADHD. Furthermore, pretesting of the survey also reflected positively on that balance. Fourth, an inherent limitation of DCE is the use of hypothetical scenarios that may or may not reflect the actual behaviour of the respondents; however, due to the nature of the study groups, there was a possibility that people may not feel comfortable when asked about their actual medication‐taking/giving practices; therefore, hypothetical choices were deemed more appropriate. It is, however, possible that different individuals may have interpreted the choice tasks differently. Although pretesting did not uncover any differences in interpretation of choice tasks, it was limited to three participants only. Furthermore, this study included participants who had experiences of taking medication to further reduce the hypothetical bias; however, it may limit the generalizability to those who do not have experience of taking ADHD medication. Finally, the sample may not be representative of the population with ADHD. For example, our sample included more females and more educated people compared to the national sample.^55^ In the case of parents, the skewed sample may not significantly affect the interpretation of findings as evidence suggests that mothers take more responsibility of their child with ADHD compared to fathers.^56^ However, in the case of adults with ADHD, it may limit the generalizability of the findings. Moreover, we relied on a self‐reported diagnosis of ADHD, which may also contribute to sampling bias.

### 4.2 Implications of findings for practice and research

Our study provides an important contribution to the literature about patient/parent preferences for ADHD and the RI that they place on various medication outcomes. Guidelines on the management of ADHD recommend that patients' and/or parents' preferences should be considered when designing treatment plans during clinical consultations.^57^, ^58^ There is a growing consensus that treatments that are more acceptable are likely to result in higher adherence, which will in turn produce improved medication outcomes.^36^ We believe that our findings provide evidence that each person has their own preferences for medication, and therefore, those preferences must be incorporated into clinical decision‐making for designing treatment plans that meet an individual's needs. Identification of individual preferences would also allow clinicians to focus on issues that are more important to the patients, provide tailored education and address their concerns, which may contribute to improved medication adherence.

## 5 CONCLUSIONS

Preferences for ADHD medication vary significantly amongst adults, and parents of children, with ADHD. Side effects were the most important factor that influenced decision‐making about ADHD medication, followed by improvement in education, social behaviour, aggressive behaviour, family functioning and stigma. There was a willingness to continue medication for the benefits of medication despite the medication having side effects. However, the extent to which a person balances the two attributes may vary between individuals. Therefore, the “one size fits all” approach to improve adherence is unlikely to be successful.

## CONFLICTS OF INTEREST

The authors declare no conflicts of interest.

## Supporting information

Supporting information.

## Data Availability

All relevant data are available in the manuscript and/or electronic Supporting Information Material.
