# Supplementary material for: Eliciting preferences for continuing medication among adult patients and parents of children with attention‐deficit hyperactivity disorder
Source: Health Expect. 2022 Mar 10;25(3):1094–107. doi: 10.1111/hex.13462 (PMC9122456; doi:10.1111/hex.13462)
Supplement: Supplementary file 1 — Supporting information. [file HEX-25--s001.docx]

**Appendix 1: Study design**

? implementation experiment

design

;alts = medA*, medB*, neither ? checking for dominant alternatives

;rows = 24

;block = 3

;eff = (mnl,d)

;alg = mfederov

;require:

neither.EDUCATION = 0,

neither.AGGRESSIVE = 0,

neither.SOCIAL = 0,

neither.FAMILYFUNCTION = 0,

neither.SIDEEFFECTS = 0,

neither.STIGMA = 0,

medA.EDUCATION + medA.AGGRESSIVE + medA.SOCIAL + medA.FAMILYFUNCTION > 1, ? Medication A needs to make some improvement

medB.EDUCATION + medB.AGGRESSIVE + medB.SOCIAL + medB.FAMILYFUNCTION > 1 ? Medication B needs to make some improvement

;model:

U(medA) = b_0[0]

+ b_education.dummy[0.001|0.002] * EDUCATION[1,2,0] ? 0 = no improvement, 1 = somewhat improvement, 2 = considerable improvement

+ b_aggressive.dummy[0.001|0.002] * AGGRESSIVE[1,2,0] ? 0 = no improvement, 1 = somewhat improvement, 2 = considerable improvement

+ b_social.dummy[0.001|0.002] * SOCIAL[1,2,0] ? 0 = no improvement, 1 = somewhat improvement, 2 = considerable improvement

+ b_familyfunction.dummy[0.001|0.002] * FAMILYFUNCTION[1,2,0] ? 0 = no improvement, 1 = somewhat improvement, 2 = considerable improvement

+ b_sideeffects.dummy[-0.001|-0.002|-0.003] * SIDEEFFECTS[1,2,3,0] ? 0 = none, 1 = mild, 2 = moderate, 3 = severe

+ b_stigma.dummy[-0.001] * STIGMA[1,0] ? 0 = no, 1 = yes

/

U(medB) = b_0[0]

+ b_education * EDUCATION

+ b_aggressive * AGGRESSIVE

+ b_social * SOCIAL

+ b_familyfunction * FAMILYFUNCTION

+ b_sideeffects * SIDEEFFECTS

+ b_stigma * STIGMA

/

U(neither) = b_education * EDUCATION

+ b_aggressive * AGGRESSIVE

+ b_social * SOCIAL

+ b_familyfunction * FAMILYFUNCTION

+ b_sideeffects * SIDEEFFECTS

+ b_stigma * STIGMA

$

**Appendix 2: Mixed logit model**

NLOGIT

;lhs=choice,cset,altij

;choices=meda,medb,none

;rpl

;fcn=med(n),educ(n),behavc(n),socc(n),famc(n),sidese(n),sidemo(n)

;pts=500;halton

;pds=nchoice

;model:

U(meda)=med+edusw*edusw+educ*educ+behavsw*behavsw+behavc*behavc+socsw*socsw+socc*socc+famsw*famsw+famc*famc+sidemi*sidemi+sidemo*sidemo+sidese*sidese+stigma*stigma+

gpedusw*gpedusw+gpeduc*gpeduc+gpbehsw*gpbehsw+gpbehc*gpbehc+gpsidese*gpsidese/

U(medb)=med+edusw*edusw+educ*educ+behavsw*behavsw+behavc*behavc+socsw*socsw+socc*socc+famsw*famsw+famc*famc+sidemi*sidemi+sidemo*sidemo+sidese*sidese+stigma*stigma+

gpedusw*gpedusw+gpeduc*gpeduc+gpbehsw*gpbehsw+gpbehc*gpbehc+gpsidese*gpsidese/

U(none)=0

$

With scaling parameter

NLRPLOGIT

    ;lhs=choice,cset,altij

    ;choices=meda,medb,none

      ;labels = bmed, bedusw, beduc, bbehavsw, bbehavc,

                  bsocsw, bsocc, bfamsw, bfamc,

                  bsidemi, bsidemo, bsidese,

                  bstigma, bgpedusw, bgpeduc, bgpbehsw, bgpbehc,

                  bgpsides,beta2d

      ;start =   -2.31202, 3.12079, 4.74974, 1.09115, 1.41835,

                  1.70924, 2.17125, .84550, 1.30417,

                  -.30705, -1.19289,-5.88729,

                  -.46174,  -1.24582, -1.29956,  .83788,  .75825,

                  -2.27131, 0

      ; Fn1 = Scaling = 1 + beta2d*SecQ

      ; Fn2 = UtilPr = bmed+bedusw*edusw+beduc*educ+bbehavsw*behavsw+bbehavc*behavc +

            bsocsw*socsw+bsocc*socc+bfamsw*famsw+bfamc*famc+bsidemi*sidemi+bsidemo*sidemo+bsidese*sidese + bstigma*stigma+bgpedusw*gpedusw+bgpeduc*gpeduc+bgpbehsw*gpbehsw+bgpbehc*gpbehc+bgpsides*gpsidese

      ; Fn5 = UtilFi = Scaling * (UtilPr )

    ?;rpl

    ;fcn=bmed(n),beduc(n),bbehavc(n),bsocc(n),bfamc(n),bsidese(n),bsidemo(n)

    ;pts=500;halton

    ;pds=nchoice

               ; Alg=BFGS

    ;model:

    U(meda)= UtilFi /

    U(medb)=UtilFi $

**Appendix 3: Mixed logit model including simulation commands**

NLRPLOGIT

    ;lhs=choice,cset,altij

    ;choices=meda,medb,none

      ;labels = bmed, bedusw, beduc, bbehavsw, bbehavc,

                  bsocsw, bsocc, bfamsw, bfamc,

                  bsidemi, bsidemo, bsidese,

                  bstigma, bgpedusw, bgpeduc, bgpbehsw, bgpbehc,

                  bgpsides,beta2d

      ;start =   -2.31202, 3.12079, 4.74974, 1.09115, 1.41835,

                  1.70924, 2.17125, .84550, 1.30417,

                  -.30705, -1.19289,-5.88729,

                  -.46174,  -1.24582, -1.29956,  .83788,  .75825,

                  -2.27131, 0

      ; Fn1 = Scaling = 1 + beta2d*SecQ

      ; Fn2 = UtilPr = bmed+bedusw*edusw+beduc*educ+bbehavsw*behavsw+bbehavc*behavc +

            bsocsw*socsw+bsocc*socc+bfamsw*famsw+bfamc*famc+bsidemi*sidemi+bsidemo*sidemo+bsidese*sidese + bstigma*stigma+bgpedusw*gpedusw+bgpeduc*gpeduc+bgpbehsw*gpbehsw+bgpbehc*gpbehc+bgpsides*gpsidese

      ; Fn5 = UtilFi = Scaling * (UtilPr )

    ?;rpl

    ;fcn=bmed(n),beduc(n),bbehavc(n),bsocc(n),bfamc(n),bsidese(n),bsidemo(n)

    ;pts=500;halton

    ;pds=nchoice

               ; Alg=BFGS

;simulate

;scenario: educ(meda,medb)=1

;arc

;full

;pwt

    ;model:

    U(meda)= UtilFi /

    U(medb)=UtilFi $

**Note:** Various scenarios were simulated as presented in the manuscript. The scenario presented in this appendix is an example of how the command was specified in the model. When one attribute-level was simulated to 1, other levels in the attribute were kept as 0.
